# Elements of Chemistry, Including the Applications of the Science in the Arts

**Published:** 1852-07

**Authors:** 


### BIBLIOGRAPHICAL NOTICES.

---

*Elements of Chemistry, including the Applications of the Science in the Arts.* By Thomas Graham, F. R. S., Professor of Chemistry in University College, London; &c., &c. *Second American, from an entirely revised and greatly enlarged English Edition; with numerous wood engravings.* Edited, with notes, by Robert Bridges, M. D., Professor of Chemistry in the Philadelphia College of Pharmacy, &c., &c. Philadelphia, Blanchard & Lea, 1852. Part I. pp. 430. Royal 8vo.

The appearance of a second American edition of this standard work on Chemistry may be regarded as, of itself, a strong evidence of its appreciation by the cis-Atlantic student. In no department of science has greater progress been made, and in none have more important facts and principles been developed, within the last quarter of a century, than in Chemistry. Especially is this remark true in reference to its application to Medicine, and chiefly, we may add, to *practical* Medicine. The time has not long passed by when Chemistry was scarcely suffered to claim allegiance with the other branches of medical science. It was looked upon merely as one of the departments of Natural Philosophy, with the leading principles and laws of which, every accomplished and well educated physician was expected to be acquainted, very much as he would be supposed to understand the principles of Mechanics or the laws of Light; but with no definite idea of its applicability to the practical part of his profession.

Well do we remember the almost universally prevalent feeling on the part of medical students in relation to the chemical lectures. Nearly without exception, was a distaste evinced for that particular branch of their studies, from their regarding it as almost an arbitrary arrangement of the schools, to be compelled to spend their time in the acquisition of a knowledge, the practical bearing of which on their future profession, they could not understand. No doubt much of this indifference of the student towards the chemical lectures was fostered by the too abstract method, then so prevalent, in which the subject was presented to him by his teachers. He would witness, perchance, the repetition of some of the brilliant experiments of Faraday on electricity, or of Melloni or Brewster on light; or he might be dazzled by the splendid proofs of the agency of oxygen gas in combustion; and even admire the beauty of the changes produced in the various decompositions of the metallic salts; but he never could realize that Chemistry constituted an integral—*indispensable* portion of his medical studies. He had, probably, a little while ago, during his collegiate term, listened to just such a course of lectures on Chemistry; and had then, as now, considered it as naturally allied to the course of mathematics or of classics, quite as much as to medicine proper.

Now, however, the case is widely different. The teacher of Chemistry has it in his power to illustrate almost every step in his course of lectures, by appeals to medical facts of the most vital importance to the physician. The so-called imponderables—Heat, Light, Electricity and Magnetism, are now shown to be most intimately concerned both in the phenomena of life, and in the treatment of disease. The experiments on the supporting power of oxygen to combustion now merely serve to illustrate and enforce the indispensable importance of that element of the atmosphere to every living being, in each act of life. The various saline and metallic compounds, the study of which formerly proved so irksome to the student, are now invested with an unexpected interest in his eyes, when they are exhibited to him as constituting most valuable agents in the treatment of disease, or as possessing poisonous or antidotal properties.

But it is chiefly in the department of Organic Chemistry that the greatest advances have been made, and the most important facts developed. It is this portion of the subject that more especially interests the medical man. Here the most important and useful contributions have been made to the science and practice of medicine. Before the splendid discoveries of the organic chemist, how little was known of the true pathology of disease, and how necessarily meagre and empirical were our therapeutics! Now, however, what a flood of light has been thrown upon diseases of the blood and urine, for example, by organic chemistry! Indeed, it is not too much to assert, that the practitioner of the present day is more indebted to the developments made in Chemistry, for correct views in pathology and therapeutics, than to any, if not to all, of the sister branches of medicine.

Professor Graham's "Elements" have been so long and so favorably known to the profession, as to require no additional commendation on our part. In the words of the American editor, "the copious selection of facts from all reliable sources, and their judicious arrangement, render it a safe guide for the beginner; while the clear exposition of the theoretical points, and frequent references to special treatises, make it a valuable assistant to the more advanced student."

The American publishers have, as yet, only issued Part I. of the work. This embraces the consideration of the laws of Heat and Light, together with a very full and complete exposition of the principles of nomenclature, and the fundamental doctrines of Chemistry under the heads of Combining Proportions, Atomic Theory, Doctrine of Volumes, Isomorphism, Isomerism, Constitution of Salts, Chemical Affinity and Polarity, (including under the latter head the subject of Voltaic Electricity,) together with the new subject of the Atomic Volume of Solids. On these principles and doctrines of the science the author is particularly clear and explicit; and he may with great advantage, as well as confidence, be consulted by the advanced student. We need hardly mention the fact that to Mr. Graham is due the discovery of the "law of the diffusion of gases;" and that we are indebted to his investigations for the interesting facts concerning phosphoric acid and the phosphates. Next in order, the simple non-metallic bodies are treated of, together with the compounds which they form with each other. Then follows the consideration of the metallic elements, which are treated of in Part I., only as far as the metallic bases of the earths, inclusive. The remainder of metals, together with the whole subject of Organic Chemistry, is reserved for Part II., which the publisher promises to have ready during the present year; and which may probably claim our notice at a future time.

The present edition is considerably larger than the former one, many portions of the work having undergone revision; and the number of illustrations is nearly doubled. The additions made by the editor are very judicious and useful; and we can most confidently recommend the work as one of the most complete and useful treatises on Chemistry with which we are acquainted.

